# Discovery and optimization of cardenolides inhibiting HSF1 activation in human colon HCT-116 cancer cells

**DOI:** 10.18632/oncotarget.25545

**Published:** 2018-06-05

**Authors:** Alina D. Nikotina, Lidia Koludarova, Elena Y. Komarova, Elena R. Mikhaylova, Nikolay D. Aksenov, Roman Suezov, Viktor G. Kartzev, Boris A. Margulis, Irina V. Guzhova

**Affiliations:** ^1^ Laboratory of Cell Protection Mechanisms, Institute of Cytology of Russian Academy of Sciences, St. Petersburg 194064, Russia; ^2^ InterBioscreen, Chernogolovka 142432, Russia; ^3^ Saint Petersburg Technical University, St. Petersburg 190013, Russia

**Keywords:** heat shock factor 1 (HSF1), Hsp70, anticancer drugs, combined antitumor therapy

## Abstract

Combinational anticancer therapy demonstrates increased efficiency, as it targets different cell-survival mechanisms and allows the decrease of drug dosages that are often toxic to normal cells. Inhibitors of the heat shock response (HSR) are known to reduce the efficiency of proteostasis mechanisms in many cancerous cells, and therefore, may be employed as anti-tumor drug complements. However, the application of HSR inhibitors is limited by their cytotoxicity, and we suggested that milder inhibitors may be employed to sensitize cancer cells to a certain drug.

We used a heat-shock element-luciferase reporter system and discovered a compound, CL-43, that inhibited the levels of heat shock proteins 40, 70 (Hsp70), and 90 kDa in HCT-116 cells and was not toxic for cells of several lines, including normal human fibroblasts. Consequently, CL-43 was found to reduce colony formation and motility of HCT-116 in the appropriate assays suggesting its possible application in the exploration of biology of metastasizing tumors. Importantly, CL-43 elevated the growth-inhibitory and cytotoxic activity of etoposide, cisplatin, and doxorubicin suggesting that the pro-drug has broad prospect for application in a variety of anti-tumor therapy schedules.

## INTRODUCTION

Traditional anticancer drugs used for chemotherapy are often inefficient because tumor cells possess a powerful protective system based on molecular chaperones [1]. The expression of molecular chaperones, many of which belong to various families of heat shock proteins (Hsps), is controlled by heat shock transcription factors (HSFs) in most cases by HSF1 [2]. HSF1 governs the function of more than 1000 genes, some of which are functionally linked to tumor progression [3]. Typical products of HSF1 activation are heat shock proteins (Hsps) Hsp27, Hsp40, Hsp70 and Hsp90. Most Hsps are involved in a cell proteostasis mechanism that corrects the consequences of improper polypeptide synthesis-modification-transport and promotes the proteolytic elimination of irreversibly damaged proteins in proteasome/lysosome machineries [4].

The majority of HSF1 molecules are maintained in an inactive state in cytosol through binding to the complex containing Hsp90, Hsp70, and Hsp40; upon heat shock, HSF1 is phosphorylated, trimerizes, and migrates to the nucleus [5]. There the factor induces the expression of certain genes by binding to their 5′-upstream DNA motifs, known as heat shock elements (HSEs) [6]. Like molecular chaperones, HSF1 promotes the survival of tumor cells [7] and fosters the growth of tumor cells in culture [3]. This fact has prompted researchers to seek HSF1 inhibitors.

The most notable of such compounds is triptolide (TPL), which has demonstrated anticancer activity in a variety of tumor models [8–9]. Despite its pronounced anticancer activity, this substance has serious toxic effects on normal cells, which limits its further clinical application [10]. Recently, compounds that directly bind to and inhibit HSF1 have been reported; some of them were also found to be toxic for several cancer cell lines [11].

The aim of this study was to search for a safer HSF1 inhibitor that would be able to sensitize tumor cells to traditional anticancer drugs and thus to reduce the dose of chemotherapeutic medicine. We selected inhibitory substances from the maximum diversity subset of the chemical library of InterBioScreen Ltd., and checked the toxicity and stability of the analogs of the successful compounds. Based on results of the screening we selected the compound that was further shown to serve as a sensitizer for several well-established anti-cancer drugs, including cisplatin and etoposide.

## RESULTS

### High throughput screening and optimization of HSR inhibitors

We used more than 1000 natural compounds and their synthetic derivatives from the collection of InterBioScreen Ltd., which represents over 600,000 chemicals (ibscreen.com). The compounds selected for screening represented randomly selected diverse subsets of chemicals including alkaloids, flavonoids, terpenoids, and sesquiterpene lactones.

**Figure 1:**
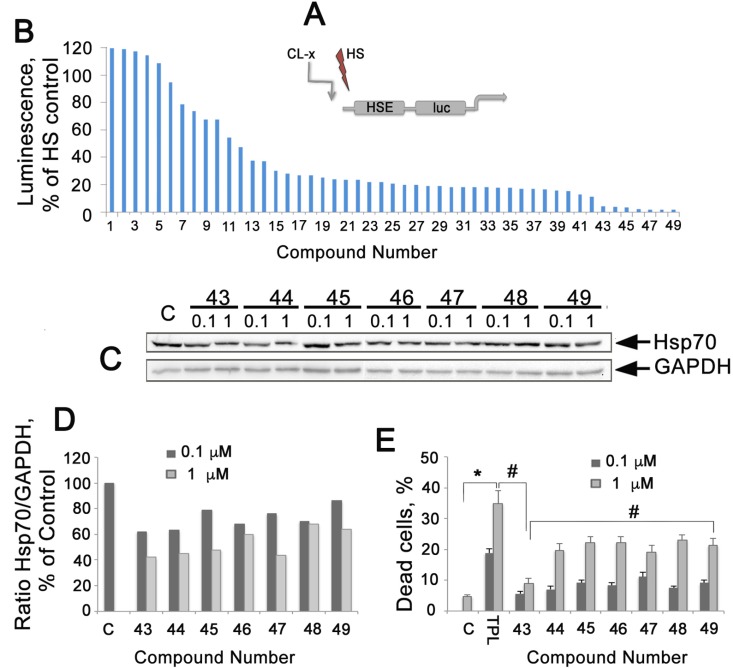
Analysis of 49 compounds analogous to CL-158 with different substituents R1-R7 on its pharmacophore, strophanthidin (**A**) Diagram of HSE-luc, a HSPs promoter reported integrated to HeLa cells. (**B**) Reporter assay of 49 analogues of CL-158. Reporter HeLa cells (transfected with plasmid bearing the luciferase gene under the HSE promoter) were seeded into wells of a 96-well plate and then heat-shocked to activate luciferase expression. Compounds at a concentration of 0.5 μM were administrated immediately after heat shock, and cells were kept at 37° C for the next 20 h before analysis. TPL was used as a reference compound. (**C**) Western blot of HCT-116 cells incubated with selected compounds at concentrations of 0.1 and 1 μM, for 20 h. (**D**) Ratio Hsp70/GAPDH intensity was evaluated as in Figure 1C. Representative data from three experiments are shown. (**E**) Toxicity of elected compounds measured as LDH activity in cell medium. ^*^p < 0,05, ^**^p < 0,01.

The screening was carried out with the use of the HSE-luciferase reporter system expressed in HeLa cells (HeLa-Luc) [12]. In this assay, HeLa cells expressed a genetic construct consisting of a heat-shock-activated luciferase gene are subjected to heat shock and simultaneously to a potential inhibitor of HSF1 (Figure 1A). One compound that was able to reduce the degree of factor activation by 63% was a substance belonged to the cardenolide (CL) family, CL-158, (see Supplementary Figure 1). However it was chemically unstable and underwent hydrolysis in water solutions. We suggested that the effect of CL-158 could be due to the activity of its hydrolytic products, ajmaline or strophanthidin. However ajmaline alone demonstrated modest activity in HeLa-Luc assay (data not shown), whereas strophanthidin suppressed HSF1 activation by 96%.

Based on the suggestion that strophanthidin may be a major pharmacophore of CL-158 and that its functional groups in different positions could provoke HSF1 inhibition differently (and consequently diminish Hsp70 expression), 49 new compounds with different substituents R1-R7 were tested (formulas are presented on Supplementary Figure 2).

Seven compounds from the second round of screening demonstrated the most pronounced HSF1 inhibiting effect on HeLa-luc assay (Figure 1B). To determine that HSF1 inhibition led to the suppression of Hsp70 expression, we employed Western blotting of HCT-116 cells incubated with the above-mentioned seven chemicals for 20 hours in two concentrations. We found that six of the seven compounds were able to dose-dependently reduce the level of Hsp70 (Figure 1C, 1D).

We analyzed the effect of all seven chemicals on HCT-116 cell viability with CytoTox96 assay and found that the compounds were toxic in the range of 7.6%-24,4% for 1 μM. The less toxic compound, CL-43, caused the death of 7.6 ± 0.5% of the cell population (Figure 1E) at a concentration of 1 μM; the calculated IC_50_ value was 479.2 ± 5.4 μM for HCT-116 cells. CL-43 was chosen for the further studies due to its high efficiency as HSR inhibitor, low toxicity and stability in water solutions.

### CL-43 inhibits the expression of molecular chaperones in HCT-116 cells and reduces their tumorigenic capacities

**Figure 2:**
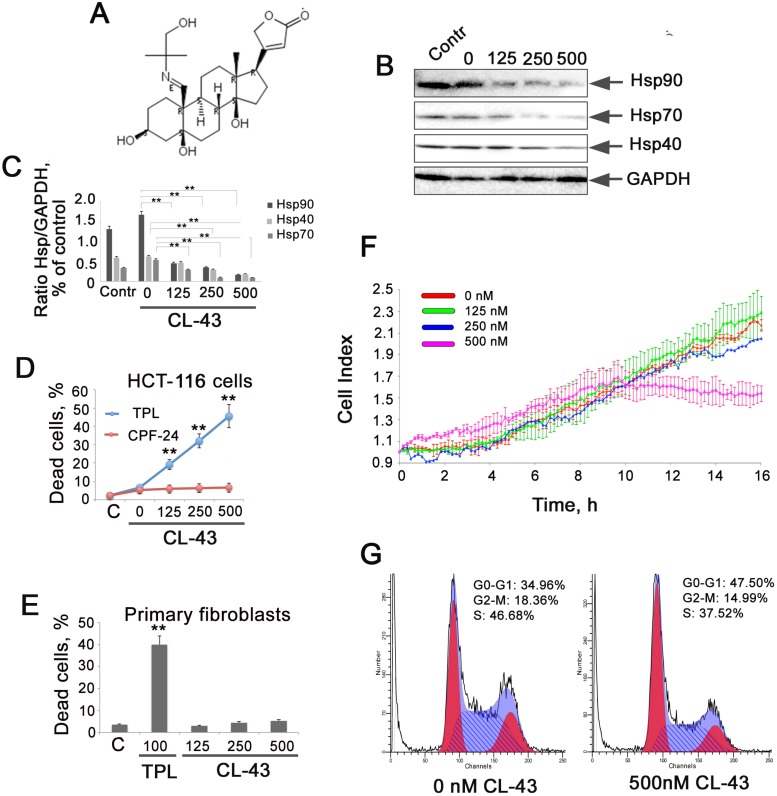
CL-43 inhibits the expression of three chaperones controlled by HSF1 and inhibits proliferation of HCT-116 cells (**A**) Formula of cardenolide CL-43. (**B**) Western blotting analysis of HCT-116 cells treated with CL-43 at concentrations of 125, 250, and 500 nM for 18 h. Point “0 nM” means cells treated with vehicle (DMSO) alone. Contr – untreated HCT-116 cells. (**C**) The intensity of bands from (B) presented as a ratio between the given chaperone and the band intensity of GAPDH used for loading control. Band intensity was estimated with use of TotalLab software summarizing the results of three independent experiments. HCT-116 (**D**) cells or primary fibroblasts (**E**) were seeded to wells of 96-well plates and then were treated with CL-43 or TPL in concentration indicated for 20 hours. The level of cell death was LDH activity in cell medium. ^**^p < 0,01. (**F**) HCT-116 cells were seeded to wells of E-plates and when they attached to the bottom, CL-43 was added in concentrations of 125, 250, and 500 nM. Recording with aid of xCELLigence equipment was started immediately after CL-43 administration and lasted 20 h. Data from five independent experiments are presented. (**G**) HCT-116 cells were treated with 500 nM CL-43 or with vehicle (DMSO) in the same volume (0 nM). After 18 h, cell cycle was measured using the flow cytometry technique.

To demonstrate that CL-43 (see formula in Figure 2A) is able to inhibit the expression of molecular chaperones controlled by HSF1, we employed Western blotting analysis. HCT-116 cells were incubated with CL-43 in various concentrations for 20 h, and after electrophoresis and blotting, the membrane was probed with antibodies against Hsp70, Hsp90, and Hsp40. The blotting data revealed that CL-43 significantly and dose-dependently reduced the content of all three chaperones. Hsp90 level was reduced by 86% when CL-43 was used at a concentration of 500 nM, while that of Hsp70 was reduced by 77% and of Hsp40 by 60%, compared to cells treated with vehicle (Figure 2B, 2C).

We have compared the toxicity of CL-43 with that of TPL in populations of HCT-116 cells and normal human fibroblasts and found that CL-43 was not harmful for both cancer and normal cells whereas TPL caused the death approximately of 50% cells (Figure 2D, 2E).

To compare the proliferating index of HCT-116 cells incubated with CL-43 in various concentrations, we analyzed the dynamics of cell growth with the use of xCELLigence equipment. The results indicated that inhibition of Hsp70 expression in HCT-116 cells led to significant reduction in growth rate only when the concentration of CL-43 was 500 nM, the population stopped growing 10 hours after CL-43 was added (Figure 2F). Since the amount of dead cells after treatment with 500 nM CL-43 was 6.1 ± 1.2% (Figure 2C), one can conclude that CL-43 in high concentrations causes growth arrest. To check this we used flow cytometry analysis of the cell cycle. Results showed that, at a concentration of 500 nM, CL-43 increased the percentage of cells at G0/G1 phase from 35% to 48%, and reduced the percentage of cells at S-phase from 47% to 37% (Figure 2G). This corroborates well with the cell index data (Figure 2F).

**Figure 3:**
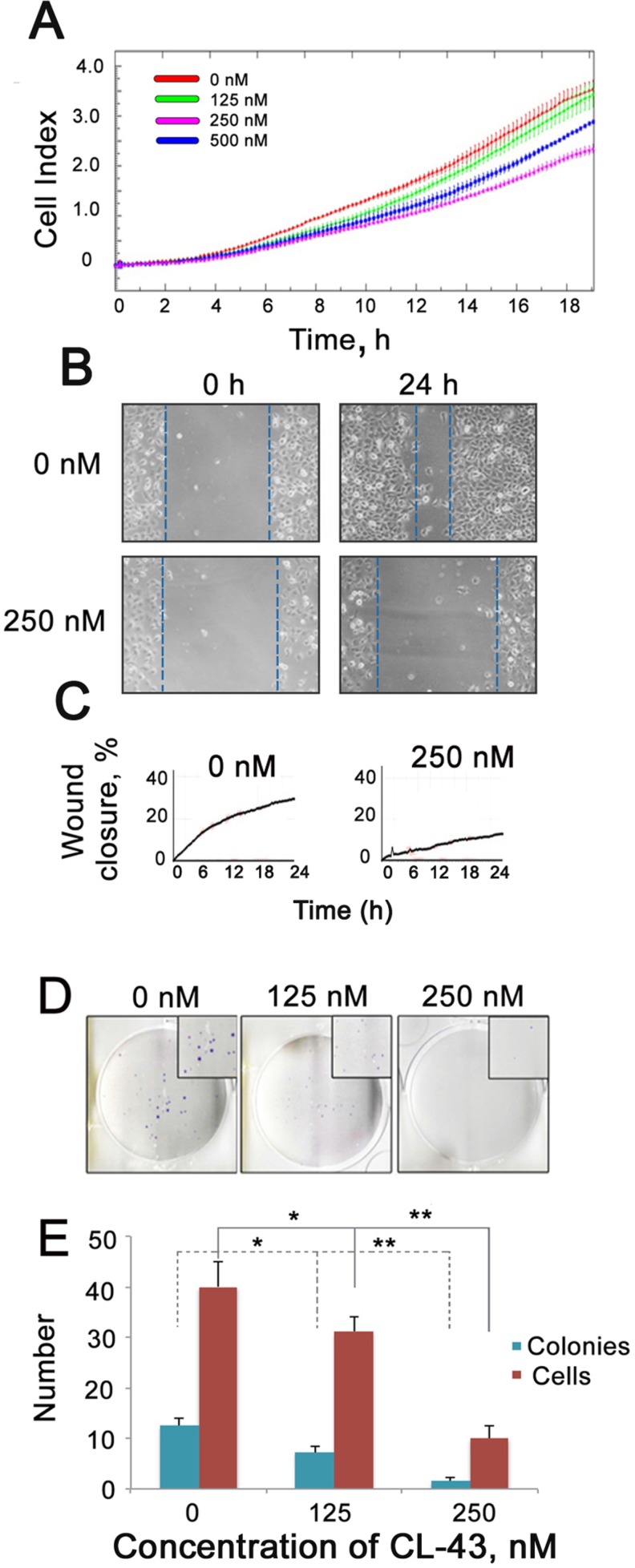
CL-43 reduces migratory and colony formation capacities (**A**) HCT-116 cells were treated with CL-43 in various concentrations for 18 h, then collected, transferred to serum-free medium and seeded into the upper wells of CIM plates of the xCELLigence system. Lower chambers of CIM plates were filled with complete medium. Recording of cells migrated through the microporous membrane of CIM plates lasted 20 h. Representative data from three independent experiments is shown. (**B**, **C**) Wound healing assay was performed with the aid of a JuLI Stage microscope. Cells were cultivated in serum-free medium for 24 h in the presence of vehicle or 250 nM CL-43 before the monolayer was scratched. Recording lasted 24 h. The wound closure was detected with microscopy (B) and monitored by JuLi Software (C). (**D**) Colony formation assay obtained with HCT-116 cells in the presence of CL-43 in concentrations of 125 and 250 nM. (**E**) The number of colonies and average number of cells in each single colony were calculated with an aid of CQ1Confocal Quantitative Image Cytometer (Yokogawa, Japan).

Microscopic observation demonstrated that the morphology of HCT-116 cells was affected by CL-43. The most remarkable change was that the cells acquired a round shape (data not shown), suggesting that they are capable of migration according to their phenotype. To test this, we first assessed the migration capacity of HCT-116 cells treated with CL-43 in various concentrations, using the CIM plate device of the xCELLigence system. CIM-plates consist of two chambers separated by a microporous membrane (pore size is 8 μm) attached to microelectrodes. In this case, the cell index calculated on the basis of the impedance measurement reflects the amount of cells migrated through the micropores. The results showed that CL-43 dose-dependently lowered the migratory capacity of HCT-116 cells (Figure 3A). Notably, the data from the wound-healing assay confirmed the above results (Figure 3B, 3C).

Another feature of metastatic cells is their capacity to form colonies and to test the effect of CL-43 we employed a colony formation assay using HCT-116 cells (Figure 3D, 3E). CL-43 significantly (*p <* 0.01) and dose-dependently reduced both colony numbers and the amount of cells in a single colony. One can conclude that treatment with CL-43 per se is not harmful to HCT-116 cells whereas affects cell cycle and proliferation; the compound was also shown to influence metastatic phenotype of the cells.

### The anti-cancer effects of combined CL-43 and conventional anti-cancer drugs

The aim of this study was to discover a minimally toxic small molecule able to suppress the protective power of cancer cells based on molecular chaperones and to increase the sensitivity of cells to anti-cancer drugs. To demonstrate that CL-43 possesses such complement activity, we treated HCT-116 cells with CL-43 in combination with several anti-tumor drugs including cisplatin, etoposide and doxorubicin. First, HCT-116 cells were treated with CL-43 in various concentrations for 20 h, and then anti-tumor drugs were added to the wells of the E-plate in the xCELLigence apparatus. The recording lasted 45 h from the time point when drugs were administrated, except for the experiment with etoposide, in which recording was stopped after 20 h of observation.

**Figure 4:**
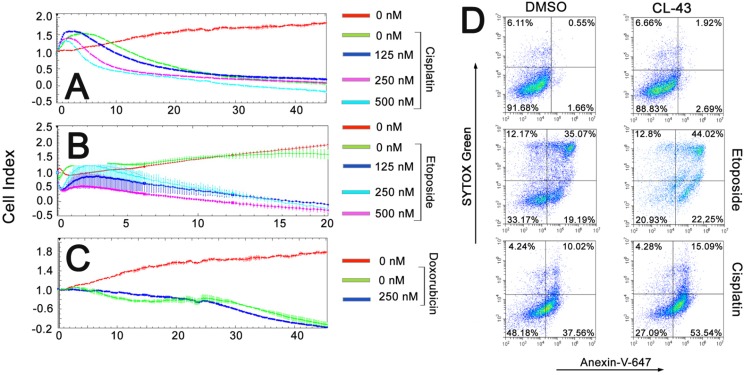
The anti-cancer effects of combining CL-43 and traditional anti-cancer drugs (**A**–**C**) HCT-116 cells previously incubated with CL-43 in various concentrations for 18 h were seeded to wells of E-plates. When the cells attached to the well bottom, anti-cancer drugs were added: (A) cisplatin, (B) etoposide and (C) doxorubicin. Recording was started immediately after drug administration and lasted 45 h (except the experiment with etoposide which lasted only 20 h). (**D**) HCT-116 cells were incubated first with CL-43 at a concentration of 250 nM, prior to etoposide (200 μM) or cisplatin (50 μM) treatment. Apoptosis was evaluated by staining cells with Annexin-V combined with SYTOX Green dye.

CL-43 was shown to be effective in all combinations, accelerating the tumor cell death caused by anti-cancer drugs (Figure 4A–4C). For example, the treatment with cisplatin alone caused the death of approximately 45% cells, whereas pretreatment of the HCT-116 cells with CL-43 at a concentration of 500 nM, increased the amount of dead cells up to 80% (Figure 4A). Contrary to cisplatin, etoposide at a concentration of 0.1 mM did not have any influence on HCT-116 cell growth and viability; pretreatment with CL-43 at concentrations of 125, 250, or 500 nM, shortened the period of cell survival and increased the death outcome in the HCT-116 cell population (Figure 4B). Even in the case of treatment with doxorubicin, which led to the death of 85% of HCT-116 cells, CL-43 impacted tumor cell sensitivity and increased the death level to 100% (Figure 4C).

Cisplatin, etoposide, and doxorubicin are well known to induce apoptosis in most cancerous cells [13], while Hsp70 and Hsp90 chaperones are able to arrest apoptosis at multiple stages of the signaling process [14]. To determine whether CL-43 may contribute to apoptosis induced by cisplatin or etoposide, we treated HCT-116 cells with the indicated drug combination and measured the level of apoptosis using flow cytometry, Annexin-V, and the SYTOX^®^ Green apoptosis staining protocol. It was found that incubation with 0.2 mM etoposide or 50 μM cisplatin caused apoptosis of 54% and 48% of the cell population, respectively (Figure 4D). Pretreatment with 250 nM CL-43 led to increases in the level of apoptosis, up to 66% for etoposide and up to 73% for cisplatin (Figure 4D).

**Figure 5:**
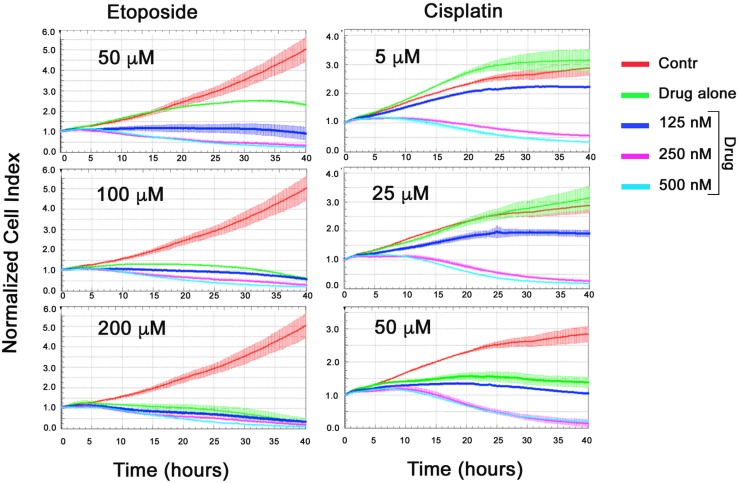
Synergistic effect of CL-43 and anti-cancer drugs HCT-116 cells were incubated with CL-43 in concentrations 125, 250 and 500 nM in combination with etoposide (50, 100 or 200 μM) or with cisplatin (5, 25 or 50 μM) added to cell culture at the same time. Recording was started immediately after drug administration and lasted 40 h.

To see synergistic effect of CL-43 and of chemotherapeutic agents we have used their combinations as it follows: 125, 250 and 500 nM of CL-43 was added simultaneously with etoposide in one of the following concentrations: 50, 100 and 200 μM; in the other experimental series CL-43 was added together with cisplatin in one of the following amounts: 5, 25, 50 μM. Cell proliferation profiles were recorded within next 40 hours using xCELLigence technology. Etoposide in low (50 μM) concentration had modest effect and the cell index was 2,5-fold higher than that in the beginning of recording (Figure 5, left column). Etoposide in the same concentration combined with 250 nM CL-43 reduced the number of HCT-116 cells by 50%. Similar growth-limiting effects of CL-43 were demonstrated for two other concentrations of etoposide, 100 and 200 μM (Figure 5, left column). Cisplatin alone in concentration 5 and 25 μM did not inhibit cell growth while the addition of 250 and 500 nM of CL-43 reduced the number of viable cells down to 50% and 27% respectively (Figure 5, right column). The data obtained with the aid of xCELLigence technology clearly showed that application of CL-43 in combination with both anticancer drugs allows to significantly reduce their dose.

### CL-43 effectively reduces Hsp70 expression in tumor cells of different origin

**Figure 6:**
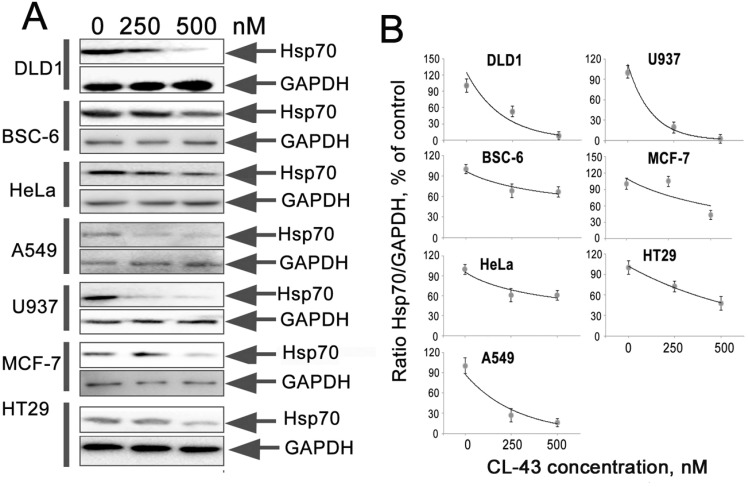
CL-43 effectively reduces Hsp70 expression in tumor cells of different origination **(A**) The cells of lines listed in the figure were treated with CL-43 at concentrations of 250 and 500 nM for 18 h and Western blotting was performed. (**B**) The intensity of bands from (A) presented as the ratio between band intensity of Hsp70 and band intensity of GAPDH used for loading control. Band intensity was estimated with use of TotalLab software summarizing the results of two independent experiments.

Finally, we assessed the efficacy of CL-43 as Hsp70 synthesis inhibitor in tumor cells of various origin. We chose seven cell lines besides HCT-116 and treated the cells with 250 or 500 nM of CL-43, or with DMSO (“0 nM”) as vehicle. The list of human cancer cell lines included two colon carcinoma cells (DLD1 and HT29), lung adenocarcinoma cells (A549), breast adenocarcinoma cells (MCF-7), cervical adenocarcinoma cells (HeLa), histiocytic lymphoma cells (U-937), and BSC-6 colon carcinoma cells recently established from human colon tumors [15]. The cells were incubated in the presence of CL-43 in the concentrations indicated, and the cell lysates were subjected to Western blotting (Figure 6A). The IC_50_ values were obtained based on results of densitometry and on the calculation of ratios between the intensity of Hsp70 and the glyceral-3-phosphate dehydrogenase (GAPDH) bands on the appropriate blot (Figure 6B). Hsp70 expression was reduced differently in various cells. IC_50_ values were 270 nM for DLD1cells, 174 nM (A549), 140 nM (U937), 425 nM (MCF-7), 410 nM (HT29), 550 nM (BSC-6) and 660 nM for HeLa cells (Figure 6B). These data demonstrate that CL-43 effectively inhibits Hsp70 in a variety of human cancer cells.

## DISCUSSION

High level of molecular chaperones in cancer cells is one of the major hurdles in clinical chemotherapy [1]. The phenomenon, known as the HSR, amalgamates the activity of a large number of genes whose products protect tumors from the deleterious effects of the microenvironment or drugs. The latter type of protection requires an increased amount of medicines, and therefore may cause multiple side effects in an organism. The success of Hsp90 inhibitors in clinical trials prompted researchers to seek drugs targeting the HSR system regulated by HSF1 [2]. The goal of the present study was to search for a small molecule able to reduce HSR-based protective efficiency and exhibiting low toxicity. The application of such a substance could reduce anti-cancer drug doses, and in doing so, reduce possible side effects.

One of such inhibitors was found to be cyclopentaneperhydrophenantrene derivative of cardenolide CL-43 which met the above criteria, (Figures 1, 2A). The compound was minimally toxic for cancer and normal cells and demonstrated the capacity to efficiently inhibit the expression of Hsp70, Hsp90, and Hsp40 basic chaperones (Figures 1E, 2B, 2C). The reduction of chaperones expression was correlated with the diminished cell growth rate (Figure 2F). This phenomenon is a well-known characteristic of cancer cells depleted of the HSR [16, 17]. Moreover, the flow cytometry data showed that the growth of a considerable portion of the population of HCT-116 cells stopped at the phase G0/G1 (Figure 2G), suggesting that, taken alone, the compound possesses growth inhibitory activity, as do most of the other pharmacological inhibitors of HSR, exemplified by KRIBB11 [18]. The latter substance was identified as the inhibitor of HSF1. Down-regulation of HSF1 in human melanoma MeWo cells with the aid of specific shRNA was associated with arrest of cell cycle on G1 phase [19] whereas TPL and another HSF1 inhibitor, phenethyl isothocyonate, induced G2/M cell cycle arrest in cancer cells [20, 21]. Knockdown of HSF1 causes reduction of Hsps content as we proved here with the use of CL-43 (Figure 2B, 2C) and this could lead to weaker association of HSP90 with its co-chaperone CDC37, finally leading to the partial depletion of the other HSP90 client kinases, Bruton’s Tyrosine Kinase (BTK), c-RAF and cyclin-dependent kinase 4 (CDK4). Treatment with triptolide or HSF1 knockdown disrupted the cytosolic complex between HSF1, p97, HSP90 and the HSP90 deacetylase- known as histone deacetylase 6 (HDAC6) [22].

Thus all ways of HSF1 down-regulation cause the suppression of cell proliferation and this phenomenon is proved for cancer cells of different origin highlighting the anti-tumor directivity of the protein inhibitors like CL-43.

Other peculiarities in cancer cell behavior were also found to be suppressed by CL-43, more specifically, migration of HCT-116 cells (Figure 3A–3C) and colony formation capacity (Figure 3D, 3E). These effects clearly resemble the consequences of HSF1 knock-down, as shown recently [23, 24]. We conclude that CL-43 by itself can reduce the growth of cancer cells and accordingly, their tumorigenicity.

Cardenolides are known from ancient Egyptians over 3000 years and have been widely used in the treatment of cardiac diseases more than 200 years [25]. During last decade cardenolides and their derivatives found an application in anti-cancer therapy [26] and therefore the fact that CL-43 possess anti-proliferation activity is in line with novel evidences on the compound versatility. Interestingly, anticancer properties of CL-43 are quite similar to other HSR inhibitors like TPL or KRIBB11 and on our mind further application of growth-limiting property of CL-43 can be directed to therapeutic schemes where such mild substances are preferable.

Since we envisioned the strategy to decrease chaperonic power of reluctant cancer cells and increase therapeutic efficacy of commonly used anti-cancer drugs we concentrated on the effects of CL-43 in combination with cisplatin, etoposide and doxorubicin. Our findings convincingly showed that in all combinations, CL-43 contributes to growth inhibition and cytotoxic activities of the above drugs (Figure 4A–4C). Notably, the compound could elevate the proapoptotic activity of etoposide and cisplatin, suggesting its possible application in combinational therapy (Figure 4D). Moreover, simultaneous adding of CL-43 and anti-cancer drugs also was effective proving synergistic effect of the compounds (Figure 5). These data correlate well with the data from groups employing combinations of classic anti-cancer drugs and chaperone inhibitors. Promising results were obtained with a combination of doxorubicin and TPL for the treatment of breast carcinoma cell lines *in vitro* and *in vivo* [27]. Another example of combinational therapy was presented by Qi and coauthors [28] who reported the upregulation of the efficiency of bortezomib by approximately 25% with KNK-437 HSR inhibitor. Comparable proapoptotic efficiency was attained in our study when HCT-116 cells were treated with cisplatin in combination with CL-43 (Figure 4A).

The mechanism of HSR inhibition by CL-43 is elusive, and we can only speculate that it is mediated by HSF1, like was shown in the case of KRIBB11, which binds to HSF1 directly [18]. The other possibility is the interference of CL-43 with the transactivation function of HSF1 without disturbing the early events of trimer formation, hyperphosphorylation, and DNA binding, typical for TPL [12].

**Figure 7:**
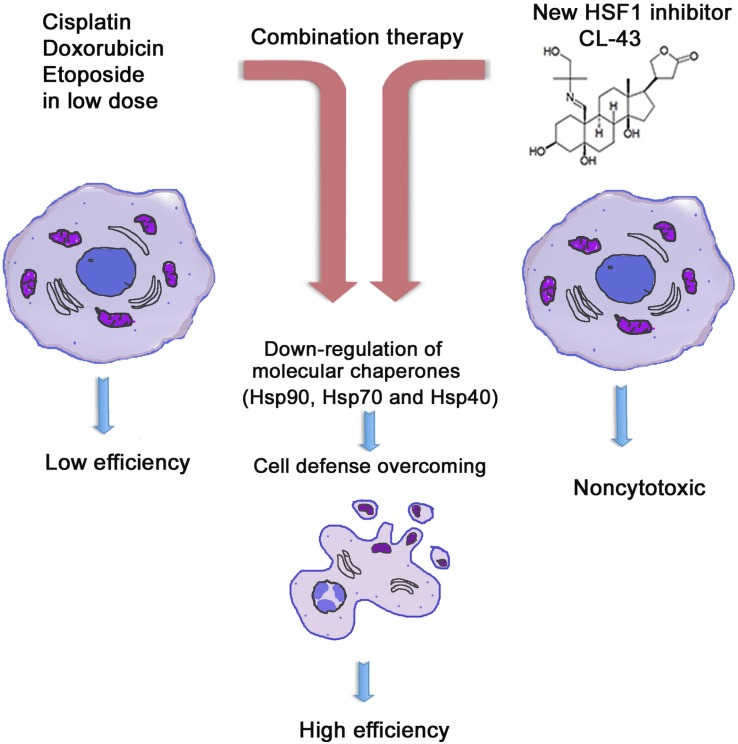
Principle of HSF1 inhibitor action in cancer cells Tumors are generally resistant to anti-cancer drugs (left panel). Since this resistance is based on HSF1 transcription factor and a few of heat shock proteins Hsp90, Hsp70, Hsp40, the combinational therapy should include compound suppressing HSF1 activity and the traditional anti-tumor medicines like etoposide or cisplatin (central panel). Newly identified compound CL-43 is safe substance that was found to elevated activity of etoposide, cisplatin and doxorubicin in cancer cells of distinct origin (right panel).

Finally, we investigated whether CL-43-mediated HSF1 inhibition is effective in cancer cells of different origin and demonstrated that the compound elicits a similar Hsp70 level-reducing effect in cells of all seven tumor lines (Figure 6). We calculated the apparent IC_50_ values of Hsp70 down-regulation and found that they ranged between 140–660 nM. Taking into account the much higher concentration of CL-43 needed to produce a cytotoxic effect, we conclude that this novel substance may serve as an efficient universal complement to anti-cancer therapy (Figure 7).

## MATERIALS AND METHODS

### Compounds

The InterBioScreen (http://www.ibscreen.com) library was employed to screen for inhibitors of Hsp70 synthesis. The chemicals were dissolved in dimethyl sulfoxide (DMSO) to obtain the desirable stock concentration, 20 mM, and stored at –20° C. To study the combined effects, we used etoposide, doxorubicin and cisplatin (all from Sigma-Aldrich, USA).

### Cell lines, antibodies and plasmids

HCT-116, DLD1, HT-29 colon carcinoma, A549 lung adenocarcinoma, MCF-7 breast adenocarcinoma, HeLa cervical adenocarcinoma, U-937 lymphoma, and HEK293T cell lines and human fibroblasts DF-2 were obtained from the Russian Collection of Cell Cultures (Institute of Cytology, Russian Academy of Sciences, St. Petersburg, Russia). BSC-6 colon carcinoma cells established from human colon tumors [15] were kindly provided by Dr. Elena Tolkunova (Institute of Cytology RAS, St. Petersburg, Russia). HeLa-luc cells were kindly provided by Prof. Richard Morimoto (Northwestern University, USA). Adherent cells, were cultured in Dulbecco’s Modified Eagle’s Medium (DMEM) supplemented with 10% FBS, 2 mM L-glutamine, and antibiotics. Primary fibroblasts were grown in DMEM/F12 and U-937 cells in RPMI media with 10% FBS, 2 mM L-glutamine, and antibiotics.

Primary mouse monoclonal anti-Hsp70 (Clone 2H9) and anti-Hsp40 (Clone J32) antibodies were produced previously in our laboratory [16, 29]. Mouse monoclonal anti-GAPDH (Clone 6C5) antibodies were kindly provided by Prof. Vladimir Muronetz (Moscow State University, Russia). Primary anti-Hsp90 was purchased from ThermoFisher Scientific (USA). Secondary peroxidase-labeled donkey anti-mouse antibodies were obtained from Jackson ImmunoResearch (USA). Plasmid with luciferase HSE-reporter was kindly provided by Prof. Richard Morimoto (North Western University, Evanston, USA).

### Luciferase reporter assay

Luciferase activity was measured with Bright Glo Luciferase kit (Promega, USA) using multichannel Fluorophot Charity (OOO “Probnauchpribor”, Russia). The measurement time was 5000 ms.

### Western blot analysis

HCT-116 cells treated with chemicals selected in the HTS (in concentrations of 50, 100, 500, and 1000 nM for 18 h) were lysed on ice in solution containing 20 mM Tris-HCl pH 7.5, 150 mM NaCl, 2 mM EDTA, 1 mM PMSF, and 0.1% Triton X-100. Equal amounts of protein were precipitated for 1 h at –20° C with acetone, dissolved in sample buffer (2% SDS, 250 mM Tris-HCl pH 7.5, 10% glycerol, bromophenol blue), electrophoresed in 11.5% polyacrylamide gel, and transferred onto a PVDF membrane using a MiniProtean System (Bio-Rad, USA). The membrane was blocked with PBS containing 5% (w/v) skimmed milk, incubated with primary and secondary antibodies at room temperature for 1 h. Band intensity was quantified using Chemidoc system (Bio-Rad, USA).

### Colony forming assay

The colony-formation test was performed according to [30], with modifications. HCT-116 cells were treated with CL-43 in concentrations of 125 and 250 nM for 20 h, washed, plated in six well plates in concentration 250 cells/wellcells/ml, and incubated for 14 days in 5% CO2 at 37° C. Then cells were fixed with 10% formaldehyde, stained with 0.01% Crystal Violet and dried. Plates were scanned with the aid of a standard scanner system (Canon MF4410, Japan).

### Wound-healing assay

HCT-116 cells were serum-deprived, and CL-43 at a concentration of 250 nM was added to the confluent monolayer. After 20 h of incubation, the HCT-116 monolayer was wounded by scratching with a 5 ml pipet tip. Wound-healing was monitored for 24 h with aid of JuLI Stage microscope (NanoEnTek, South Korea) and monitored by JuLi Software.

### Lactate dehydrogenase (LDH) assay

Lactate dehydrogenase (LDH) assay was used to determine the toxicity of the selected compounds. HCT-116 cells were seeded in 96 well plates and treated for 20 h with compounds in various concentrations. Cell culture medium was then collected and LDH activity was detected with the aid of a Cytotox96 Non Radio Cytotoxity Assay Kit (Promega, USA) according to the manufacturer’s recommendations.

### Cytotoxicity assay with xCELLigence system

The xCElligence system (ACEA Biosciences) provides noninvasive and label-free monitoring of cell viability and growth in real-time, based on measurement of the electrical impedance of cells adhered to an electrode on the well bottom. Increased impedance indicates that an increased number of cells is adhered to the bottom at this time [31]. HCT-116 cells were placed in 16 well E-plates (ACEA Biosciences, USA) at a concentrationof 40000 cells/ml. After 18 h, cells were treated with CL-43 in various concentrations for 20 h, and then etoposide (100 µM), doxorubicin (5 µM) or cisplatin (50 µM) were added to cells. Cell proliferation was then monitored for 48 h using the RTCA xCELLigence System. Data analysis was performed using RTCA Analysis Software.

### Detection of apoptosis

Detection of apoptosis was performed with the aid of Annexin-V TM 633 (Life Technology, USA) combined with SYTOX^®^ Green dye (Life Technology, USA) staining. HCT-116 cells were treated with CL-43 at a concentration of 250 nM (20 h), alone or in combination with anti-cancer drugs etoposide (200 μM) or cisplatin (50 μM) 20 h later, cells were collected, washed in cold PBS, resuspended in the binding buffer provided by the manufacturer, and stained with Annexin-V-Alexa647 and SYTOX^®^ Green (Life Technology, USA) according to manufacturer’s recommendations. The cell cycle was then measured with the aid of the CytoFlex Flow FACS (Beckman Coulter, USA) using laser with λ = 488 nm and analyzed with ModFit LT (Verity Software House Inc, USA) software.

### Cell cycle analysis

Cells were seeded into a six-well culture plate at 1.3 × 10^5^ cells/ml and treated with CL-43. After 20 h of incubation with the compound, harvested cells were washed three times with cold PBS and fixed in 96% ethanol at 4° C for 20 min. Cells were then treated with 10 μg/mL RNase and stained with 50 μg/mL Propidium Iodide (PI) for 30 min at room temperature in the dark. The cell cycle was then measured with the aid of the CytoFlex Flow FACS (Beckman Coulter, USA) using laser with λ = 488 nm.

### Statistics

The data are reported as the mean ± standard errors of the mean (SE) of at least three independent experiments. One-way ANOVA test supplemented with posthoc test was used to calculate the statistical significance of the experimental data and the level of significance was set as *p <* 0.05 (^*^) or *p <* 0.01 (^**^). The growth curves obtained in proliferation and migration experiments were exported Form the RTCA software as mean Cell Index (CI) value. The CIs for particular stage of growth curves were read and statistically analyzed directly from the RTCA software that uses a Student’s *t*-test. Data were normally distributed.

## SUPPLEMENTARY MATERIALS FIGURES



## Abbreviations

FBS: fetal bovine serum
HSE: heat shock element
HSF: heat shock factor
Hsp: heat shock protein
GAPDH: glyceraldehyde 3-phosphate dehydrogenase
TPL: triptolide
