# Supplementary material for: Discovery and optimization of cardenolides inhibiting HSF1 activation in human colon HCT-116 cancer cells
Source: Oncotarget. 2018 Jun 5;9(43):27268–79. doi: 10.18632/oncotarget.25545 (PMC6007471; doi:10.18632/oncotarget.25545)
Supplement: Supplementary file 1 [file oncotarget-09-27268-s001.pdf]

# Discovery and optimization of cardenolides inhibiting HSF1 activation in human colon HCT-116 cancer cells

## SUPPLEMENTARY MATERIALS

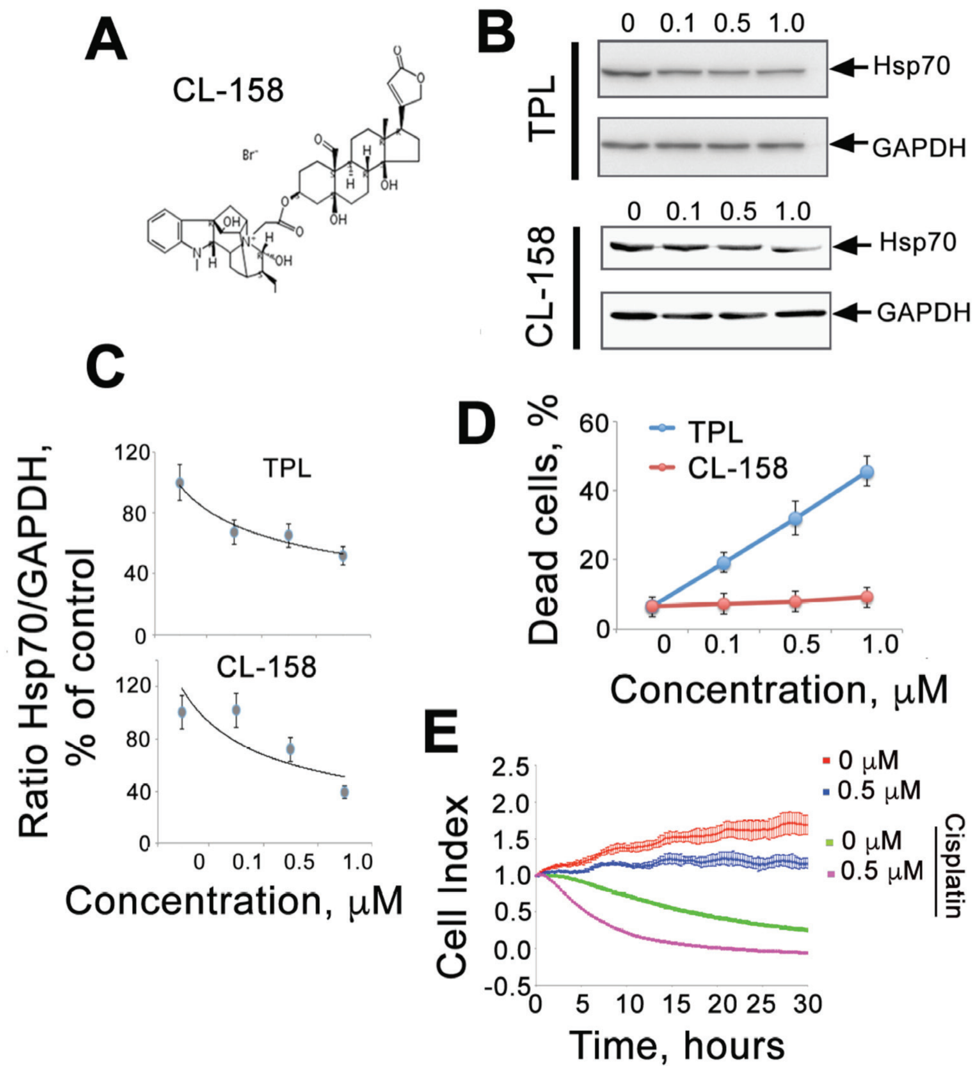


**Supplementary Figure 1: Search for HSF1 inhibitors from the InterBioScreen library.** (**A**) Formula of CL-158 which was selected from the first screening of 1000 compounds from the chemical library and demonstrated inhibitory activity comparable to triptolid (TPL).The data were obtained with the aid of reporter HeLa-luc cells bearing plasmid with the luciferase gene under the HSE promoter. (**B**) Western blot of HCT-116 cells incubated with CL-158 and TPL in concentrations indicated. (**C**) The intensity of bands from (B) presented as a ratio of the band intensity of Hsp70 to the band intensity of GAPDH used for loading control. Band intensity was estimated using TotalLab software to summarize the results of three independent experiments. (**D**) Toxicity of CL-158 and TPL measured as LDH activity in cell medium. Representative data from three experiments are shown. (**E**) Proliferation rates of HCT-116 cells incubated with cisplatin alone or in combination with CL-158. HTC-116 cells were incubated first with CL-158 at a concentration of 0.5 μM for 18 h, and then with cisplatin (50 μM). Recording was started immediately after cisplatin administration and lasted 30 h.

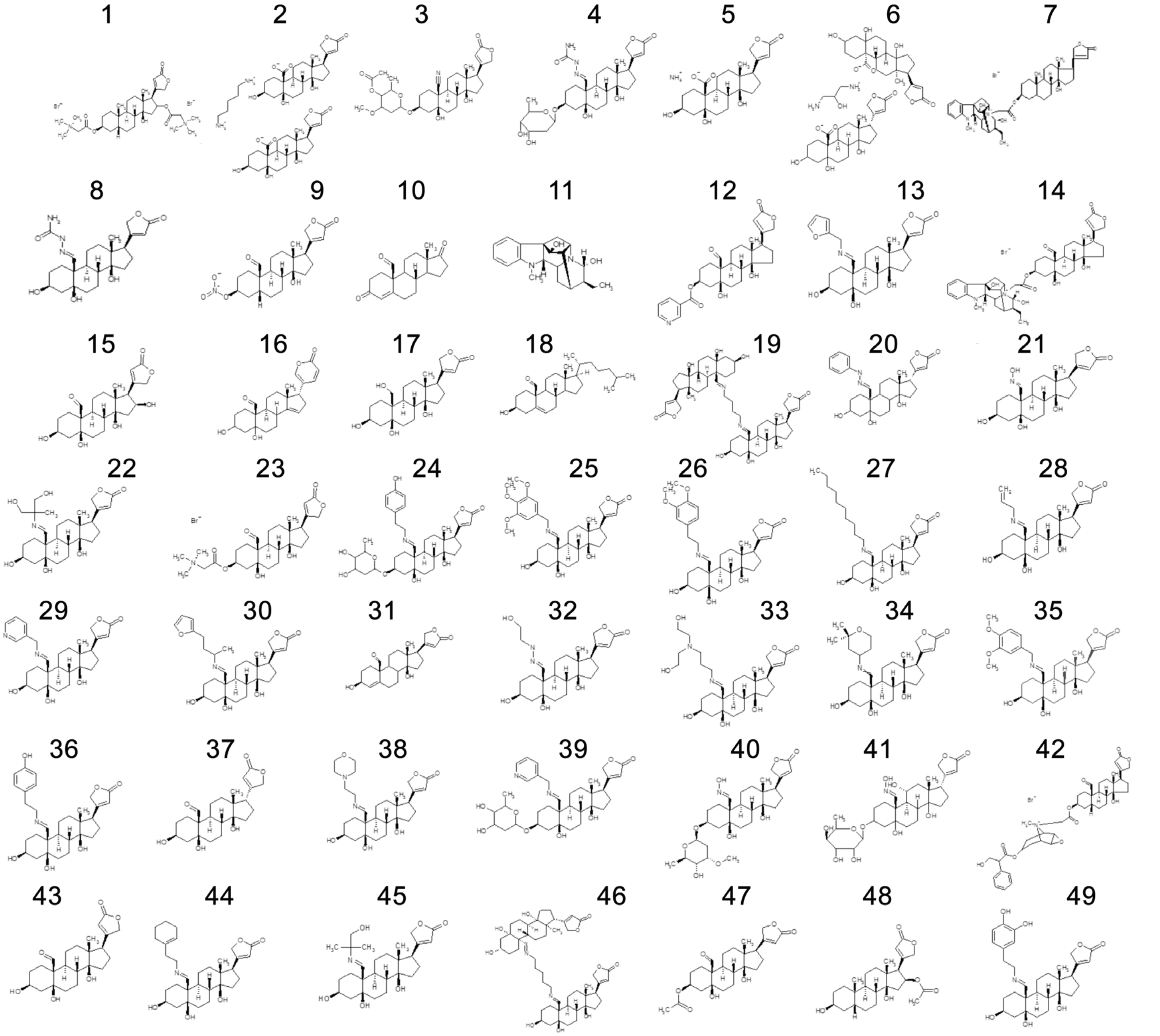


**Supplementary Figure 2: Formulas of 49 analogues of CL-158 compounds with different substitutes R1-R2 on its pharmacophore, strophandin.**
